# Analyzing the Effect of Time in Migration Measurement Using Georeferenced Digital Trace Data

**DOI:** 10.1215/00703370-8917630

**Published:** 2021-02-01

**Authors:** Lee Fiorio, Emilio Zagheni, Guy Abel, Johnathan Hill, Gabriel Pestre, Emmanuel Letouzé, Jixuan Cai

**Affiliations:** Department of Geography, University of Washington, Seattle, WA, USA; Max Planck Institute for Demographic Research, Rostock, Germany; Asian Demographic Research Institute, Shanghai University, Shanghai, China; and Wittgenstein Centre (IIASA, VID/ÖAW, WU), International Institute for Applied Systems Analysis, Vienna, Austria; Department of Geography, University of Washington, Seattle, WA, USA; Data-Pop Alliance, New York, NY, USA; Data-Pop Alliance, New York, NY, USA; and Massachusetts Institute of Technology Media Lab, Cambridge, MA, USA; Department of Geography and Resource Management, The Chinese University of Hong Kong; and Wittgenstein Centre (IIASA, VID/ÖAW, WU), International Institute for Applied Systems Analysis, Vienna, Austria

**Keywords:** Migration, Mobility, Big data, Methods

## Abstract

Georeferenced digital trace data offer unprecedented flexibility in migration estimation. Because of their high temporal granularity, many migration estimates can be generated from the same data set by changing the definition parameters. Yet despite the growing application of digital trace data to migration research, strategies for taking advantage of their temporal granularity remain largely underdeveloped. In this paper, we provide a general framework for converting digital trace data into estimates of migration transitions and for systematically analyzing their variation along a quasi-continuous time scale, analogous to a survival function. From migration theory, we develop two simple hypotheses regarding how we expect our estimated migration transition functions to behave. We then test our hypotheses on simulated data and empirical data from three platforms in two internal migration contexts: geotagged Tweets and Gowalla check-ins in the United States, and cell-phone call detail records in Senegal. Our results demonstrate the need for evaluating the internal consistency of migration estimates derived from digital trace data before using them in substantive research. At the same time, however, common patterns across our three empirical data sets point to an emergent research agenda using digital trace data to study the specific functional relationship between estimates of migration and time and how this relationship varies by geography and population characteristics.

## Introduction

Issues of data availability and comparability have long hampered quantitative migration research. Missing or incomplete data is a common problem, and inconsistencies in how migration is defined by institutions can make migration estimates from traditional sources such as population censuses, administrative records, or survey data difficult to synthesize across context (de Beer et al. 2010; Rogers et al. 2003). Faced with a persistent lack of comprehensive migration data, demographers and other social scientists have begun to draw on new sources of digital data for the study of migration. Among the promising new sources are digital trace data (Deville et al. 2014; Frias-Martinez et al. 2012; Hawelka et al. 2014). Generated as a byproduct of everyday information technology use, digital trace data consist of individual-level records of digital behavior, which may include information on a person’s physical location (Menchen-Trevino 2013). With the global proliferation of digital technology, digital trace data are increasingly common and are available in a wide range of forms that are potentially useful to migration scholars, such as metadata associated with cellular calls and texts, GPS information captured passively by smartphone applications, and geotags posted to social media or other location-based social networks (LBSNs) (Girardin et al. 2008).

However, in their raw, unprocessed state, locational digital trace data do not correspond to any meaningful measure of migration. Instead, they represent millions upon millions of high-resolution locational traces, each of which is a record of a unique individual at a particular place at a particular time. This fundamental characteristic of digital trace data both poses challenges and offers new opportunities for research. There are no standards or best practices for how to convert highly granular locational trace information into estimates of migration transitions or events. Not all moves are migrations. Thus, how researchers choose to operationalize the largely ambiguous distinction between migration and other kinds of movement (e.g., long-distance commuting, tourism, seasonal travel) will greatly affect the consistency of migration estimates generated from digital trace data. Although it is well-established that these new forms of data come with bias, linking digital trace estimates of migration with traditional survey or administrative estimates is not necessarily the only validation strategy. As we demonstrate in this paper, much can be learned about the bias and coverage of these data by systematically assessing the consistency of migration estimates with respect to definition parameters.

Although analyzing digital trace data poses methodological challenges, the unique granularity of these data also provides researchers with a novel opportunity to address substantive questions about the spatial and temporal dimensions of migration phenomena. Much of this work is well underway. For example, research on internal migration has historically been limited by inconsistent and arbitrary subnational administrative geographies, such as state or province borders that bisect metropolitan regions or important rural-urban gradients contained within a single administrative region (Niedomysl et al. 2017). By mining for patterns in individual mobility traces or activity spaces, researchers have developed a number of approaches that exploit the high spatial resolution of digital trace data to reveal more meaningful spatial scales that define different kinds of movement (Jones and Pebley 2014; Palmer et al. 2013). But as we argue in this paper, the granularity of digital trace data is not limited to *spatial* granularity. The temporal definitions used in migration research can also be inconsistent or arbitrary. Thus, much can be learned about the temporal scales that define certain kinds of movement by leveraging the *temporal* granularity offered by these new kinds of data.

In this paper, we offer a general framework for converting digital trace data into migration estimates. Conceptually coherent and simple to implement, the goal of the framework is to produce migration estimates that follow the logic and structure of migration *transition* data—that is, to estimate the share of individuals in a population who undergo a transition from one place of residence to another over a given time interval.

When implemented systematically, our framework has two applications. First, it can be used to evaluate the consistency of migration estimates derived from a given source of digital trace data. Second, provided that the digital trace migration estimates are deemed to be of sufficient quality, this framework can be used to investigate substantive issues related to population movement and time. The strategy in both applications is to generate many different estimates of migration from the same data source and then to assess how the estimates vary with respect to a quasi-continuous time parameter.

This kind of analysis should look familiar to demographers because it resembles a survival function—that is, the proportion of a population who become migrants (or remain nonmigrants) with increasing time—and it can be used to test simple hypotheses. Migration researchers have long theorized that migration rates should go up with increased exposure to the risk of moving (Rees 1977). A five-year rate is almost always higher than a one-year rate in surveys that collect migration data using both intervals. However, by systematically applying our framework to digital trace data, we can produce many intermediate estimates and empirically investigate the cumulative effect of exposure to the risk of migration for different populations and geographies. This kind of approach is novel in migration research. Moreover, if we find that our migration estimates are inconsistent with respect to time (e.g., if a migration flow between two regions defined by a span of 12 months differs considerably from the flow defined by the same 12-month span *plus two weeks),* we can consider this evidence that our data and our definition of migration are producing problematic estimates and that we need to do more to identify and parse out short-term moves (e.g., travel) in the estimation procedure. We show how this can be done by adjusting the temporal place of residency criterion.

In the sections that follow, we begin by outlining the conceptual difficulties involved in defining migration with respect to time as well as the related challenges that can arise when studying migration with digital trace data. We introduce our framework for converting digital trace data into migration transition data and discuss our hypotheses. To provide a heuristic against which to compare our empirical results, we propose a simple stochastic model and evaluate its properties with micro-simulation. We then apply our method to three unique data sets in two internal migration contexts: call detail record (CDR) data in Senegal, and Twitter and Gowalla data in the United States. Finally, we briefly demonstrate one way that our method could be used to compare and contrast the geographic patterns of short-term mobility and long-term migration flows.

Because digital trace research is novel and data coverage remains an issue, we do not attempt to draw definite substantive conclusions from our findings. Instead, by applying our method to three data sets of varying quality from different contexts, we focus on the kinds of insights that this framework can reveal. Nevertheless, our findings should be of interest to researchers involved in developing standards for inferring migration from digital trace data and in deepening our understanding of the spatial and temporal dimensions of migration phenomena and migration measurement. In this era of continuously changing and increasingly heterogeneous spatial-temporal patterns of population movement, it is unlikely that the data problems migration researchers face will be resolved easily. However, when analyzed using the framework introduced in this paper, digital trace data can provide timely insights at a level of detail and with a degree of flexibility that can greatly improve efforts to infer migration patterns and advance our understanding of complex migration phenomena.

## Background

Studying migration entails measuring the movement of people in space and time. However, distinguishing migration from other kinds of mobility can be difficult and ultimately depends on the purpose of the measurement. In terms of space, defining migration is complicated by the varying social, political, and economic meanings of the different geographic units between which people move. International migration is important for political reasons and is simple to conceptualize as a relocation across a national border. However, internal migration is more common globally and can have causes and effects similar to those of international migration (Ellis 2012; King and Skeldon 2010). The task of identifying meaningful geographies of migration at subnational scales is not straightforward, and comparisons of internal migration patterns across contexts are often hampered by differing standards (Bell et al. 2015; Long et al. 1988).

In terms of time, defining migration is complicated by the varying frequencies with which people move. People may engage in return or onward migration or move around for short periods as evidenced by the growing phenomena of circulation and short-term or temporary mobility (Hannam et al. 2006; King 1978; Rogers 1995). There is no theoretically grounded definition of permanence (Williams and Hall 2002). Even individuals who have lived away from their country of birth for many years might someday return (Cassarino, 2004), and increasing numbers of people split their time between multiple locations (Gössling et al. 2009). To determine when a person becomes a migrant, rather than temporary visitor, governments often rely on a length-of-stay criterion, such as 12 months. However, such criteria are arbitrary and differ from context to context.

Scholars of migration have long been aware of the complexities of measuring migration, but their ability to systematically investigate this issue has been constrained by a lack of high-quality longitudinal data. Because most survey data are cross-sectional, these data can generally be used to estimate migration at no more than one or two intervals (e.g., the place of residence at the time of the survey is compared with the reported place of residence one year ago or five years ago). This limitation has the effect of masking the degree and the character of short-term mobility and repeat migration behavior.

In some cases, researchers have used panel survey data to study the characteristics of repeat migrants. For example, using data from the Panel Study on Income Dynamics, DaVanzo (1983) found that migrants with higher levels of education are less likely to return and more likely to move onward to a third location. Although panel survey data are valuable for understanding cohort and life course migration dynamics, they typically cannot be used to provide estimates of population-level migration trends because of their small sample sizes. Administrative data such as address registries or tax records can be used to investigate the spatial and temporal dynamics of migration measurement. For example, Goldstein (1964) used Danish registry data to demonstrate that repeat migration behavior is common among young adults. More recently, Weber and Saarela (2019) used linked registry data from Finland and Sweden to show that many moves among young adults are temporary and short term. In general, however, access to population registry data is difficult to obtain, and not all countries keep accurate administrative records.

## Challenges of Measuring Migration With Digital Trace Data

Given the limitations of traditional migration data, georeferenced digital trace data are a boon to migration scholars because of their size, simple structure, and high spatial and temporal resolution. These data come from many sources and have become increasingly abundant with the growing adoption of telecommunication technologies across the globe. Digital trace data can be collected actively when people post their location using LBSNs, such as Yelp, Foursquare, or Instagram; or passively when people use telecommunication technology to make calls, send text messages, or use smartphones and web applications. The structure of these data—that is, tuples consisting of individual ID, timestamp, and location—is the same regardless of the platform or service from which they originate, and the levels of spatial and temporal detail that they provide afford researchers a high degree of flexibility in deciding how to measure migration. However, using digital trace data in migration research poses conceptual challenges that reflect both the longstanding definitional issues discussed earlier and the indirect manner in which the data are collected.

### Defining Place of Residence

An obvious problem that can arise when estimating migration using georeferenced digital trace data is that these data contain no direct information on an individual’s place of residence. Unlike a survey (which might ask respondents about their residential history) or an administrative records system (which requires individuals to register each change of address), digital trace data simply log the location of an individual at a particular moment in time. Without further context-specific information, it is difficult to determine whether the position of a given individual corresponds to place of residence. Thus, to determine whether an individual is a migrant, it is first necessary to make some inferences about where the individual typically spends time.

In recent years, published studies have provided techniques for inferring residency from georeferenced digital trace data. Gonzalez et al. (2008) used a sample of 100,000 mobile-phone users to demonstrate the regularity with which most individuals spend time at home and work. These regular patterns made it possible to assign individuals to well-defined areas. The authors offered one method for doing so: namely, summarizing the location of each individual during a specified period and calculating radius of gyration and center of mass defined by their movement trajectory. Others have taken this idea further, attempting to use digital trace data to generate separate inferences regarding an individual’s home, work, and other ancillary locations. For example, to estimate the home location of individuals from a sample of 3 million mobile-phone users in Singapore, Jiang et al. (2017) restricted each individual’s set of positions to those occurring at night (from 7 p.m. to 7 a.m.). Then, having linked each mobile-phone user to a home location, the authors demonstrated how to identify different kinds of daily mobility trajectories, ranging from staying home to moving between several places throughout the city.

The decision about how locational information should be summarized depends on the quality of the data and the research objectives. Although researchers would ideally have access to information about the daily activity spaces of individuals in a given sample, this may not be feasible if individuals are irregularly observed or if the geographic information is coarse, as is the case with many social media–generated geotags (Stock 2018). Moreover, for the purposes of migration research, daily activity information is often unnecessary and can make the desired migration behavior more difficult to parse. Not all administrative units are arbitrary, and there are ways to make use of the spatial and temporal granularity that these new data provide without using them to estimate activity spaces. Nevertheless, because digital trace data contain no direct information on an individual’s place of residence, researchers will always need to group multiple observations to infer the place of residence regardless of whether the strategy is to map activity spaces or simply to assign each individual to their most frequented administrative unit.

### Issues With Coverage Bias

The more commonly discussed challenge that can arise when using digital trace data is coverage bias. The penetration of various digital technologies and platforms is uneven. In many cases, digital trace data are not accompanied by additional demographic information. Moreover, it can be difficult to assess the one-to-one relationship between a user ID number and an individual. Cell-phone sharing is common (Blumenstock et al. 2010), and social media data can contain bots and business accounts. Complicating matters further, digital trace data can also produce biased estimates of migration if the platform or service through which they are generated is associated with certain kinds of mobility behavior. For example, Bojic et al. (2015) showed that because geotags from Flickr (a photo-sharing social media platform) tend to capture travel and vacation activity, it is difficult to use these data to accurately identify the user’s home location.

Separating issues of bias with respect to digital trace data from the conceptual ambiguities that complicate migration measurement, however, is important regardless of the data source. Even if it were possible to obtain digital trace data containing the accurate location of every individual at every minute of the day, a person’s migration behavior would not be self-evident. In this scenario of total surveillance, there would still be a need to apply methods, rooted in migration theory, to determine which kinds of mobility behavior in the data meet which definitions of migration.

A potential upside of digital trace data is that they can still be useful in migration research even if they are of lower quality. For many applications, the individual-level accuracy of digital trace data is arguably less important than the population-level migration signal. The field of demography has a long history of working with biased or incomplete data, and as we argue in the following section, the bias of a given digital trace data set can be evaluated by assessing the extent to which it produces migration estimates that are consistent.

## Conceptual Framework and Method

In this section, we introduce our framework for converting digital trace data into estimates of migration. The overarching goal of the framework is to isolate three interrelated but distinct temporal dimensions that define a migration transition estimate: the *start* or reference point, the temporal *buffer* or residency window, and the *interval* or exposure period. By systematically altering each dimension, researchers can assess the consistency or sensitivity of the migration estimates generated from a given digital trace data set. Based on migration theory, we develop two simple hypotheses for how we expect migration estimates to behave. Testing these hypotheses has applications for both data quality assurance and substantive analysis. Before we introduce the framework, we explain our decision to estimate migration transitions.

### Event Data and Transition Data

In the migration literature, researchers make a distinction between event data and transition data. Event data consist of information pertaining to relocations that have occurred over a given period. Transition data consist of information on the population that has relocated over a given period (Rogers et al. 2010). As we have discussed, unprocessed digital trace data meet neither of these definitions. Each observation is simply a record of an individual in a specific location at a specific time. Because transition data are more commonly estimated in survey data and used by demographers to study population change (Haenszel 1967), we propose a framework for estimating transitions. Going forward, unless otherwise specified, a *migration* refers to a transition, and a *migrant* refers to a person who has transitioned. A *migration rate* refers to the proportion of people who have migrated.

We estimate transitions rather than events to simplify the conceptual scope of the problem. Estimating a transition involves determining whether an individual’s place of residence at time *t* is different from place of residence at some interval *u* in the future. By making this our goal, we avoid trying to determine *how many* migrations have occurred within a digital trace data set over a specified period. If an individual moves more than once over an interval, a given estimate of migration will capture one move at most. If this individual is a return migrant, having moved away and come back over the interval, then we would count that person (incorrectly) as a nonmigrant. Our argument is that by varying the specification—producing many migration estimates with a range of intervals—we can assess the effect of return migration on the population-level migration signal.

### The Start, Buffer, and Interval Approach

**Fig. 1:**
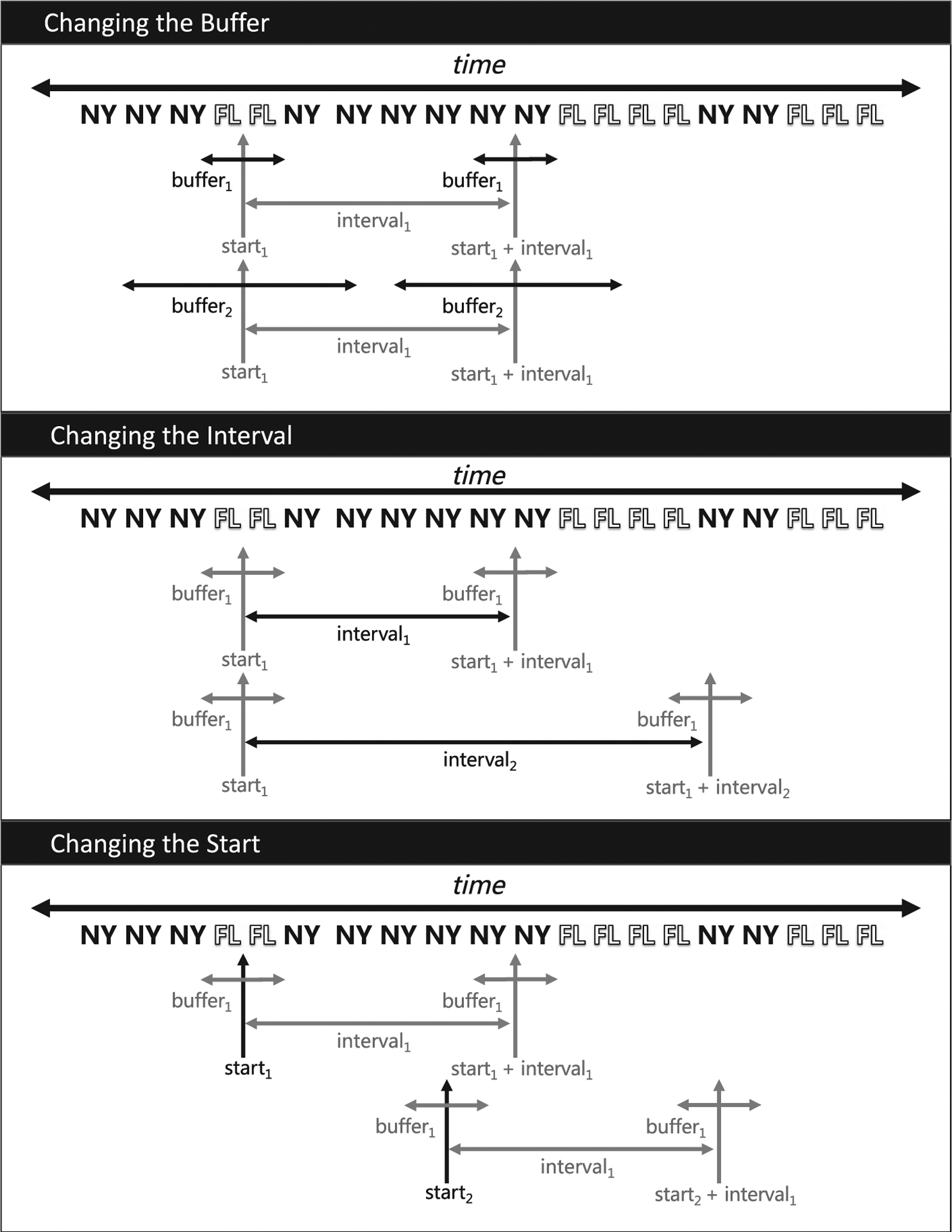
Three interlocking but distinct dimensions of migration measurement: start, buffer, and interval. By changing one while holding the other two fixed, we can assess how migration estimates are affected by seasonality (start), residency criteria (buffer), and cumulative exposure to migration risk (interval).

The logic of our framework is simple. First, we specify a reference date or *start.* Then, for all individuals in the data, we infer the person’s residency for some specified window or temporal *buffer* around the start. Next, we select as a second reference date some specified period or *interval* in the future and infer the person’s residency around that date using a temporal buffer of the same size. Finally, with estimates of each individual’s place of residence at two distinct points in time to compare—one at the start and the other at the end of the interval—we determine whether the individual is a migrant or nonmigrant. Figure 1 illustrates the implementation of our framework to a hypothetical timeline corresponding to an individual who travels back and forth between two U.S. states, New York (NY) and Florida (FL). In the top row of each panel, the specification of the start, the buffer and the interval are the same. In the bottom row, we show how one dimension can be changed while the other two are held fixed.

The strength of this framework is its flexibility. As long as digital trace data exist for a population and period of interest, many different estimates of migration can be calculated by systematically changing the start-buffer-interval specification. The only rule that must be followed when using this approach is that the interval must be of greater length than the buffer size. If, for example, we want to estimate the number of transitions over a three-month interval, we cannot use six months of data to estimate the place of residence at the beginning and the end of the interval. To do so would result in a double counting of observations and a conflation of the effects of interval and the buffer size. Nevertheless, by isolating three distinct temporal dimensions of migration measurement, our framework makes it possible to assess the consistency of estimates generated from a given set of digital trace data in conceptually coherent way: (1) the start measures the effects of seasonal or period trends; (2) the buffer measures the sensitivity to temporal residency criteria; and (3) the interval measures the effects of exposure to the risk of migrating.

### Evaluating the Consistency of Digital Trace Migration Estimates

Now that we have established how our framework can be used to generate many different estimates of migration from the same set of digital trace data, the question becomes how to evaluate the output. Our strategy is to analyze migration estimates in a manner equivalent to that of a survival function. This strategy involves assessing how the number of estimated migrants changes as the *interval* changes. In other words, we identify the specific set of individuals observed living in place *i* at reference point *t* and then evaluate the proportion of individuals in this population who have left place *i* as the interval, *u*, increases. This simple analytic strategy leads us to propose two interrelated hypotheses about regularities in migration measurement.

### Consistency and Interval

First, we expect to find that migration estimates increase as the interval increases. The logic behind this expectation is straightforward. As the population that resides in a particular place is exposed to the risk of migrating, the number of people who migrate away should also increase. Although this hypothesis might seem so obvious as to be of little value, testing it on a given set of digital trace data is useful for assessing data quality. If the data coverage is poor or if the underlying behaviors that generate the data are biased toward other kinds of mobility (such as travel), then the migration estimates will likely be irregular with respect to interval. Thus, instead of observing a slow increase in the rate of migration, we might see sharp spikes or a multimodal trend line as the migration signal is obscured by periodic short-term mobility and returns.

Moreover, if the digital trace data are deemed to be of sufficient quality, then analyzing the relationship between the migration estimates and the interval will provide useful and novel information for characterizing migration dynamics. Although migration estimates have long been theorized to increase with increased exposure to the risk of moving, empirical data on the precise functional relationship between migration estimates and intervals are scarce. Based on data from surveys that estimated migration using both a one-year and a five-year interval, migration scholars have observed that the relationship is nonlinear: that is, five-year estimates tend to be higher than one-year estimates but are not five times as high (Kitsul and Philipov 1981; Rees 1977). Moreover, considerable variation in the relationship between one- and five-year estimates has been observed across contexts (Rogers et al. 2003), and evidence of inconsistencies between the spatial structure of migration measured with one-year and five-year intervals suggests that there are different patterns of return and onward migration (Rogerson 1990). By leveraging the temporal granularity of digital trace data, our framework can provide insight into this so-called one-year/five-year problem in migration estimation.

### Consistency and Buffer

Second, we expect to find that migration estimates are higher and more inconsistent when they are produced with smaller temporal buffers. The logic behind this expectation follows that of the first. Measuring migration transitions using digital trace data requires us to infer each individual’s place of residence at two points in time and then to determine how many individuals have relocated over the interval. If we use a very small window of time on either side of the interval to infer each individual’s place of residence, we would expect to capture both short-term moves (i.e., tourism; long-distance commuting; or travel for work, education, or family) and long-term moves (i.e., migration) in our estimate. This would make the estimate higher than if we use a larger buffer size to screen out short-term moves (Bell 2004). Moreover, because short-term moves are characterized by return behavior—it is only a short-term emigration if the person comes back—we expect to find that small buffer estimates are multimodal with respect to the interval. For example, the number of people observed at a location other than their place of residence might spike during a holiday period and then decline as most of these people return to their place of residence when the holiday is over.

This hypothesis might seem self-evident, but it also has valuable applications for assessing data quality. As researchers have begun exploring the use of digital trace data in migration research, a common validation goal has been to compare digital trace estimates with traditional survey or administrative estimates of migration. However, the best way to produce comparable estimates has yet to be established. For example, if a survey conducted on March 1, 2015, asked respondents where they currently reside and where they resided one year prior, producing a similar digital trace estimate for validation would entail inferring the place of residence on March 1, 2014, and March 1, 2015, for each individual in the data set. Although this may seem straightforward, it is unclear how much data on either of the intervals is needed to sufficiently screen out short-term moves occurring around those two dates. Using only the locational information from one day at either end of the interval—March 1, 2014, and March 1, 2015—would likely be insufficient. Would it be better to use a week? Two weeks? A whole month? The answer to this line of inquiry will depend on the quality of a given data set, and we argue that investigating the relationship between temporal buffer size and the consistency of migration estimates will help make these kinds of determinations.

Studying the effect of the buffer size also provides a useful framework for evaluating different residency criteria and residency inference methods. As we stated previously, because of the fundamentally atomistic nature of digital trace data, we can make inferences about each individual’s place of residence only by grouping some of her observations. This is true regardless of whether the geography of residence is predefined (e.g., national borders) or mined from the data (such as an activity space, established by individual commuting trajectories). The simplicity of the buffer concept means that any number of functions can be used to infer residency *within* a buffer, or to compare buffers to assess whether a migration has occurred. For example, following Roseman (1971), we could extract the spatial polygon defining each user’s activity space within a buffer and then identify migrants as those whose activity spaces at either end of the interval fail to overlap. How migration estimates vary with respect to buffer size can provide information on how different residency inference methods perform. It is possible, for example, that with only one day’s worth of information on either end of an interval, most methods for inferring residency will perform the same. However, as the buffer size is increased and more data is incorporated in the inference procedure, the differences between methods should become more pronounced. Because the quality of digital trace data can vary considerably, this kind of analysis would be useful for justifying a particular analytical approach.

## Research Questions

Our two research questions extend from our discussion of interval and buffer size. These research questions, which are simple and easy to evaluate, represent the primary application of our framework.

- *Research Question 1: Do migration estimates increase as the interval increases?* We expect to find that the number of people who migrate from their place of residence will increase as the interval increases because of their added exposure to the risk of migration. Although the strength of this relationship should diminish at long intervals because of return migration, we expect to observe a largely positive relationship between the interval and the migration estimate.
- *Research Question 2: Do migration estimates decrease and become more consistent as the buffer size increases?* We expect to find that the number of people who have migrated from their place of residence will decrease as buffer size increases. With larger buffer sizes comes a larger amount of data that can be used to infer location at either end of the interval, increasing our ability to accurately estimate long-term relocations by screening out short-term moves.

How a particular digital trace data set performs with respect to these research questions will provide useful information on the suitability of the data for migration research. Moreover, once it has been established that the data are of sufficient quality, studying the specific relationship between migration estimates and interval or buffer size will deepen our understanding of the temporal complexities of migration phenomena and their measurement. Empirical data on how migration estimates change with respect to a quasi-continuous time interval could be used to address the one-year/five-year problem by allowing researchers to chart the specific functional relationship between population-level migration behavior and exposure to the risk of migrating. At the same time, empirical data on how migration estimates change with respect to buffer size could be used to evaluate different techniques for inferring residency and provide a basis for further analysis of the relationship between patterns of short-term mobility and patterns of long-term migration.

Answering these research questions does not, however, entail validating digital trace migration estimates using traditional estimates or assessing bias using some external source of more trusted data. Although this kind of validation can be seen as an essential component of any substantive digital trace migration research, we argue that it unnecessary for the purposes of demonstrating the utility of our framework. This argument rests on two points. First, given the lack of standards governing how highly granular digital trace data should be converted into migration estimates, we suggest that evaluating the consistency of such estimates should be considered a *preliminary* step to validating with traditional survey or administrative estimates. Only after it has been established that a given data set produces consistent estimates should an attempt be made to link these data to any other kind of data. Second, we expect that most digital trace data biases would not hamper our ability to validate the *internal* consistency of the migration estimates they produce. For example, even if the users on a particular social media platform are disproportionately young, we would still hypothesize that estimates of their migration activity will increase as the interval increases. It is for this latter reason that we apply our method to simulated data and empirical data of varying quality and from different contexts. Although we expect that both of our hypotheses will be confirmed using these different kinds of data, precisely how well they perform is a key insight that will be provided by the application of our framework.

## The Simulation Model

Having outlined our framework and our research questions, we present a simple simulation model to produce data that will meet our stated expectations. The goal of the model is not to simulate a specific context or replicate precise patterns in our empirical data. Instead, the purpose of the model is to explore, using simple behavioral assumptions, how short-term mobility and migration might be manifested within individual-level time-and-place data. The simulated data will provide a point of reference against which we can evaluate the patterns observed in the empirical data introduced in the next section.

### Strategy

We simulate data that take the form of tuples (individual ID, timestamp, and location). The structure of each tuple is simple and mimics the format of the locational digital trace data discussed in this paper. A single tuple does not provide much information about where a given individual resides. However, a series of these tuples over time for the same individual offers insights into patterns of residency and into patterns of mobility and migration between different places. The underlying assumption of our model is that each individual has a latent characteristic: namely, a home location (such as a U.S. state) that conditions the individual’s mobility behavior. Individuals will be observed most often in their home locations; however, individuals who are observed away from their homes for a sufficiently long period may be assumed to have changed their home locations.

In our approach, we simulate timelines for a population of *m* individuals. Each individual has known location *l* at each unit of time 1, 2, … , *t* such that individual *i* can be represented by a vector:

$$\left\{l_{i,1},l_{i,2},\;\ldots \;,l_{i,t}\right\},$$

where *l*_*i*,*t*_ is the location of individual *i* at time *t*.

We then build a model in which units of time are equivalent to one week (i.e., an individual is observed only once per week), with only two possible locations, 1 or 0. The probability that individual *i* is observed at either 1 or 0 at time *t* is represented by a simple Bernoulli random variable conditional on the individual’s “home” attribute, which can also only be 1 or 0. This gives us two conditions:

$$P\left(l_{i,t}|\text{home}\;=1\right)=\{\begin{array}{ll} p, & \text{ for }l_{i,t}=1\\ 1-p, & \text{ for }l_{i,t}=0 \end{array}$$

and

$$P\left(l_{i,t}|\text{home}\;=0\right)=\{\begin{array}{ll} p, & \text{ for }l_{i,t}=0\\ 1-p, & \text{ for }l_{i,t}=1 \end{array}.$$

Although the decision to use independent Bernoulli random trials to model short-term mobility rests on strong assumptions, we chose this method for its simplicity. Empirical evidence has demonstrated that the duration of temporary moves skews heavily toward shorter lengths of time (Bell 2004), and that the probability of observing consecutive Bernoulli values reasonably replicates the smaller likelihood of taking extended trips (e.g., three months) relative to taking shorter trips (e.g., one week). In future research, more realistic distributions of short-term mobility could be inferred directly from the empirical data. But given that here we are using our model only as a heuristic for evaluating our empirical data, we argue that relying on a Bernoulli distribution will suffice for now.

To model long-term relocation, we add an additional feature. If an individual is observed “away” from “home” for *k* consecutive weeks, then the probabilities associated with being observed in the location designated as “away” become those previously associated with being in the location once designated as “home”:

$$\text{ if}\;l_{i,t+1}=\ldots =l_{i,t+k}=0|\text{home}\;=1,\;\text{then}\;0\to \text{home}$$

$$\text{if}\;l_{i,t+1}=\ldots =l_{i,t+k}=1|\text{home}=0,\;\text{then}\;1\;\to \;\text{home}.$$

For example, take a scenario in which we observe a set of individuals for whom the probability of being home in a given week, *p,* is equal to .7, and the threshold of relocation, *k,* is equal to 4. If we observe these individuals for 100 weeks, then the rate of transition should be approximated by the probability of a streak of four or more consecutive weeks away occurring in 100 Bernoulli trials. This value can be obtained using recursion with the following formula:

$$S(N,K)={\left(1-p\right)}^{K}+\sum_{j=1}^{K}{\left(1-p\right)}^{j-1}\left(p\right)S\left(N-j,K\right),$$

where (1 − *p*) is the probability of being observed away from home on a given week, *S(N,K)* is the probability of being observed *K* or more consecutive weeks away from home out of *N* weeks, and *j* is the position of the first week an individual is observed at home (Greenberg 1970). Either we observe an individual away from home *K* consecutive weeks in the first *K* weeks (which has the probability (1 − *p*)^*K*^), or we observe the individual at home at least once in the first *K* weeks (at position *j*). In the latter case, the probability of going away for *K* or more weeks is equal to the probability of doing so following the *j*th week. Using the values from our example, this formula returns a value of .433.

### A Simulated Outcome

Continuing with the preceding example, we simulate 1,000 individual timelines with the probability of being home on a given week, *p*, equal to .7; and the long-term move threshold, *k,* equal to 4. Each individual is observed for 100 weeks. We then derive many different migration rates from the simulated data by systematically changing the start-buffer-interval specification. As we would do when using empirical data, we estimate that a simulated individual is a migrant if place of residence at the start of the interval is not the same as place of residence at the end of the interval. In this case, we infer the place of residence by calculating the modal location—either at home or away from home—during the buffer. If there is a tie, we take the first location to hit the maximum.

**Fig. 2:**
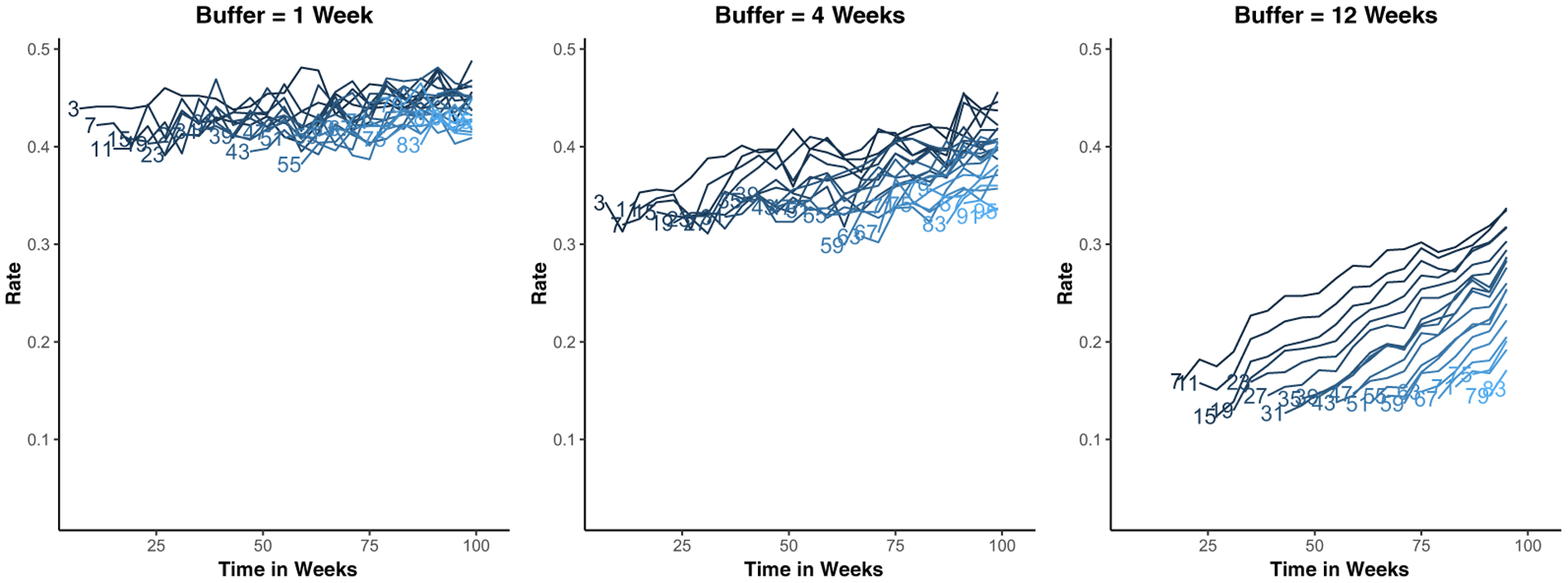
Migration rates derived from simulated data. Each line tracks the rate of individuals classified as migrants as the interval increases from a specific reference point, while the buffer is held fixed.

In each of the three panels in Figure 2, we track how the migration rate changes as the interval increases, while holding the buffer fixed at one of three different values: 1, 4, or 12 “weeks.” The *y*-axis is the proportion of movers— the migration rate—and the *x*-axis is time. A line represents a set of rates derived using a common start, which, when followed left to right, tracks the proportion of migrants as the interval grows. (For a schematic, refer to the right-hand panel of Figure 1.) The lines are plotted such that their position over the *x*-axis corresponds to the date associated with the end of the interval, and shading indicates start date, with later starts being darker. The start value is also plotted at the base of each line.

Figure 2 illustrates how we expect migration estimates to vary as we systematically change the buffer and interval size. When the buffer is small, at either 1 (left panel) or 4 (center panel), the observed rates of migration are high and multimodal. We set the conditions of our model (*p =* .7) such that individuals exhibit a high degree of short-term mobility. Although the overall rates appear to increase slightly as the interval increases, the rate of long-term relocation is somewhat masked by the short-term noise. When the buffer is increased to 12 (right panel), the signal associated with short-term mobility is mostly removed. Very few individuals are observed away from home more than 6 times in 12 tries unless they have relocated; thus, less short-term return migration is observed. In this plot, the trend lines start lower but rise consistently as the interval increases. Taken together, the three panels of Figure 2 illustrate what we expect to find in our empirical data. As the buffer size increases, the high levels of migration and mulitmodality resulting from short-term mobility are reduced. Thus, we see a consistent, positive relationship with respect to interval.

## Data

The empirical portion of the analysis is conducted on three data sets: call detail records (CDR) in Senegal from the telecommunication company Orange-Sonatel; and two sets of social media data in the United States, one from Twitter and other from Gowalla. In this section, we describe the three data sets and how the differences between them could affect our estimation. Because a major motivation for developing our framework is to take advantage of the *temporal* granularity of digital trace data, we downplay the potential uses of the spatial granularity of these data in our subsequent demonstration and analysis. However, as we noted previously, any number of sophisticated spatial techniques could be used within a buffer to infer location. For all three data sets, we infer the place of residence by simply assigning individuals to the administrative unit in which they most frequently observed—their modal administrative unit. In the case of a tie, we assign the individual to the first administrative unit to achieve the maximum level observed.

### Orange-Sonatel

Our analysis makes use of anonymized CDRs obtained via the Orange-Sonatel 2014 Data for Development (D4D) Challenge. These data consist of phone calls and SMS exchanges between more than nine million Orange-Sonatel customers in Senegal between January 1, 2013, and December 31, 2013. The specific data set used in this paper is a subset of the CDRs corresponding to a random sample of 150,000 subscribers. Only the 146,352 users meeting the following criteria were included in the sample:

1. Users having interactions on more than 75% of days in the given period.
2. Users having had an average of fewer than 1,000 interactions per week (given that users with more than 1,000 interactions per week were presumed to be machines or shared phones) (de Montjoye et al. 2014).

**Fig. 3:**
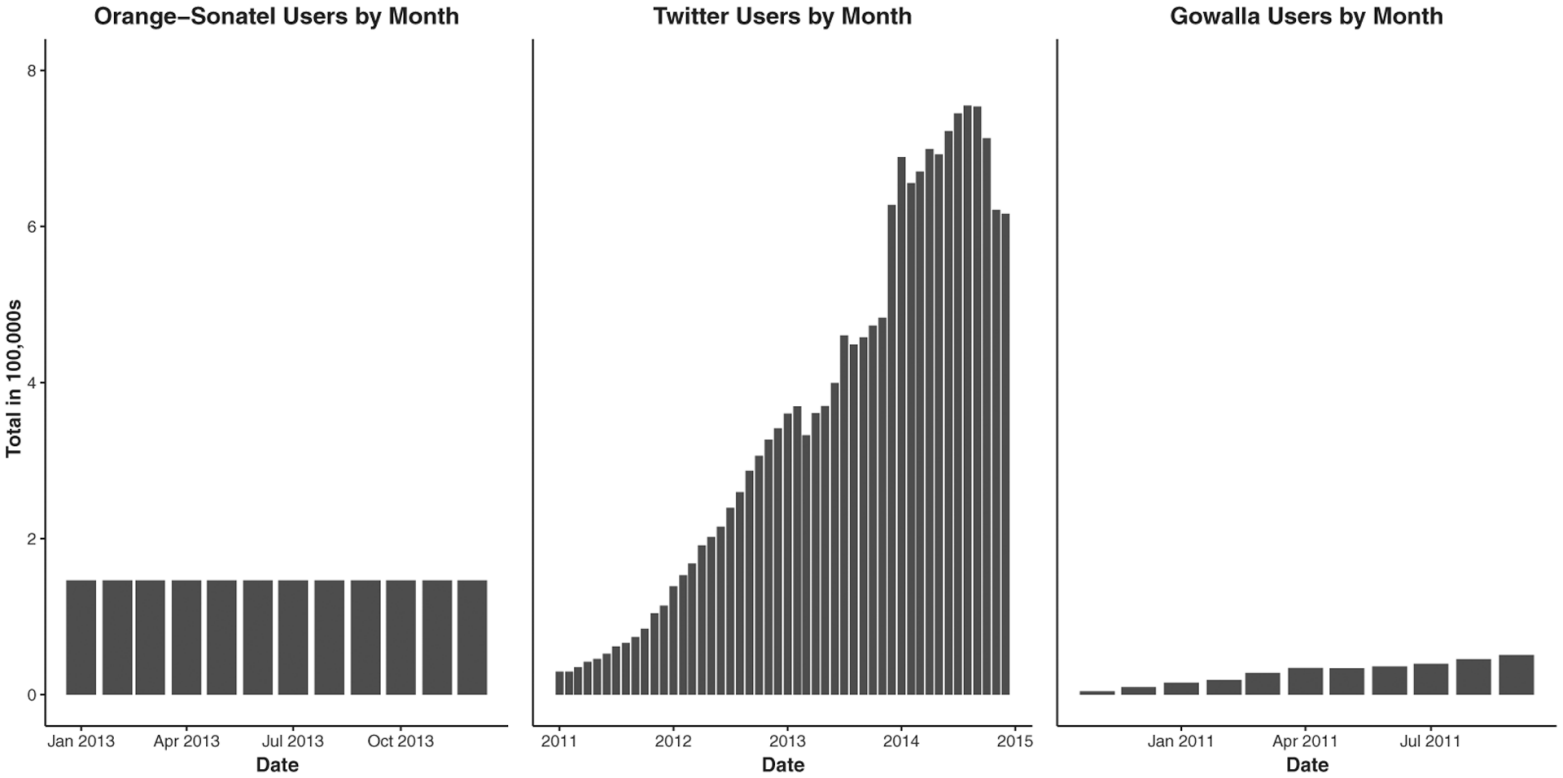
Counts of unique users by month in the Orange-Sonatel, Twitter, and Gowalla data sets

The data consist of roughly 561 million CDRs representing all the interactions (voice call or SMS) placed or received by these users in the 2013 calendar year (Figure 3). Each record provides a numerical pseudonym representing the user, the timestamp of the call, and the *arrondissement* (third administrative level) in which the user was located when the interaction took place. The D4D data set divides Senegal’s territory into 123 *arrondissements,* which can be grouped into 45 *départements* or 14 *régions.* The latter, the 14 *régions* of Senegal, are the geographic units used in this paper. We define migrant as an individual who changes *régions* over a given interval. Because these administrative units are so small, especially relative to the U.S. administrative units used in the Twitter and Gowalla analysis, we may expect much higher estimates of migration and mobility in Senegal. On the other hand, the consistency with which the cell-phone users are observed might make the estimates derived from this data set more stable.

### Twitter

We also analyze a large set of Twitter data extracted from a long-term archive of the 1% Twitter stream sample (Archive.org 2016). Only Twitter accounts with at least one tweet geotagged within the United States between January 2011 and December 2014 are included. Because of top-down changes Twitter made in early 2015 to the kinds of locational information contained within tweets, the data collected after 2014 are not directly comparable and are therefore excluded from the analysis (Tasse et al. 2017). We use the latitude and the longitude associated with each tweet to place it in one of nine U.S. Census divisions,^1^ and we define migration as a change in residency with respect to these divisions.

The Twitter data consist of roughly 447 million geolocated tweets from 1.9 million Twitter users spanning 2011–2014 (Figure 3). The mean number of geotagged tweets per user is 267, but the users are not necessarily observed every week. On average, a user’s tweets appear in 24 weeks, spread over a range of about one year. Sporadic tweeting means that under certain migration specifications, some user timelines will exhibit either left- or right-censoring. In cases in which data are missing at either or both ends of an interval given a particular buffer, the user is excluded from both the numerator and the denominator for that particular migration estimate. Unlike for the Orange-Sonatel CDR data, we make no effort to limit our sample to those individuals who were consistently observed over the period. Instead, we take a maximalist approach to see how well our hypotheses hold up when our framework is applied to a very imperfect data set. Thus, we are interested in determining the extent to which we can use increased buffer size to screen out short-term mobility.

### Gowalla

Finally, we analyze geolocated data captured through the check-in feature of the mobile geosocial network Gowalla. Similar to the more familiar check-in app Foursquare, Gowalla was a short-lived platform on which users shared their locations. For each post, Gowalla stored user ID information, a timestamp, and latitude/longitude coordinate data—sufficient information for estimating how many users changed location over time. These lesser-known Gowalla data have also been used by other researchers, including Cho et al. (2011), who used the check-ins to conduct an analysis of short-term mobility and social networks. This data set includes 6,442,890 check-ins generated from 107,092 unique users between November 2010 and October 2011. The mean number of check-ins per user is 60. On average, a user’s check-ins appear in nine weeks, spread across three months. As with the U.S. Twitter data, we use U.S. Census divisions as the geographic unit of analysis, and we estimate internal migration that occurred between divisions. Like for the Twitter data, we make no exclusions based on how consistently the users are observed in the data set. Instead, we are interested in determining whether our hypotheses still hold when applied to a small, noisy data set.

## Empirical Results

In the following section, we first compare broad patterns from our empirical results with the patterns from our simulation model. We then describe the differences between the empirical results. Finally, we briefly discuss how short-term mobility estimates might be used to indirectly infer long-term migration.

### Comparison With Simulated Results

**Fig. 4:**
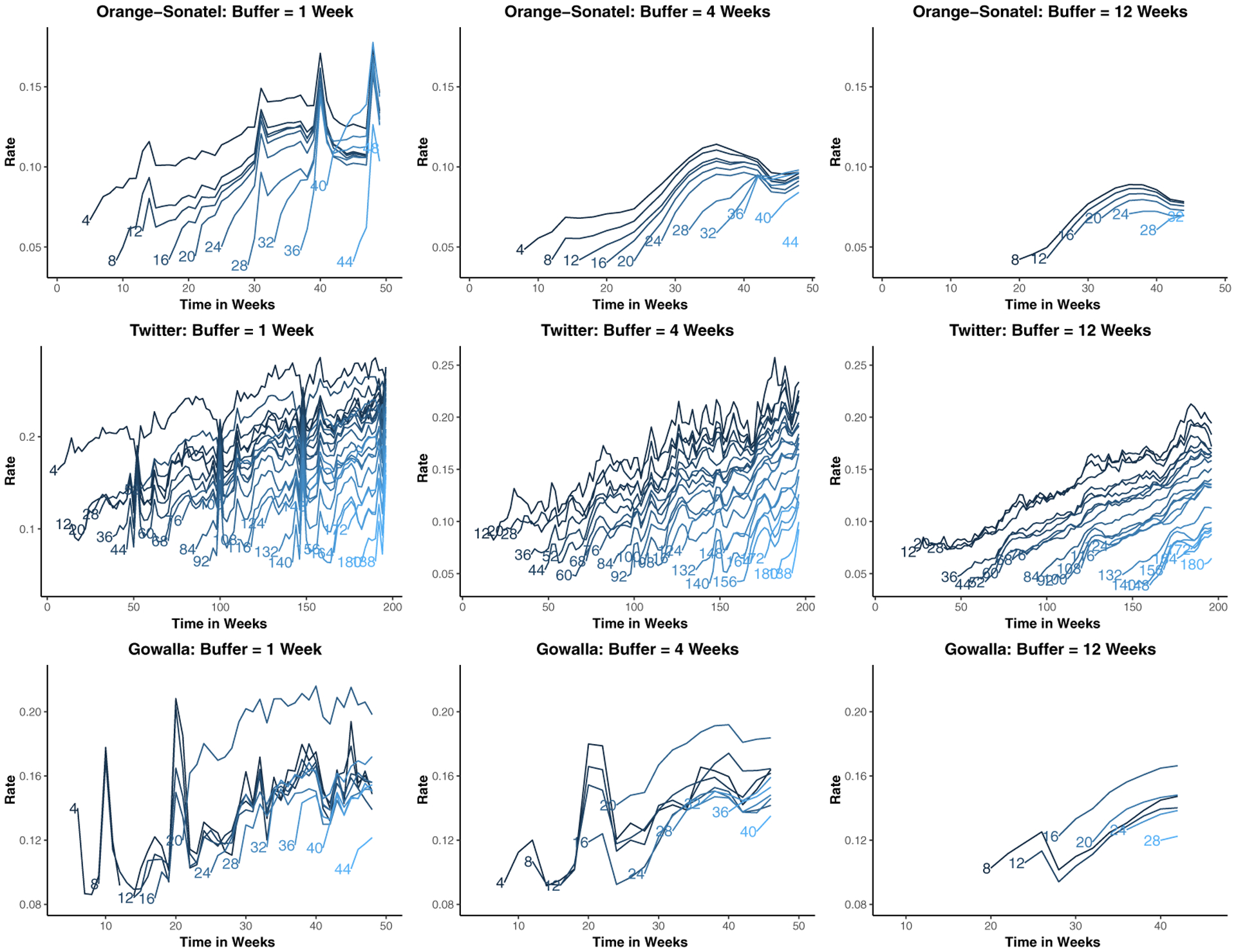
Estimates from the three empirical data sets of change in the rate of migration with increases in the interval from a specific start, while the buffer is held fixed at 1 week, 4 weeks, and 12 weeks

We perform the same start-buffer-interval analysis on our empirical data that we previously performed on our simulated data. Results are presented in Figure 4. The rows correspond to the three data sets: CDRs from Orange-Sonatel in Senegal; geotagged tweets from Twitter in the United States; and Gowalla check-ins, also from the United States. Each column shows a set of migration estimates produced while holding the buffer fixed at 1 week, 4 weeks, or 12 weeks, respectively. In each panel, the *y*-axis is migration rate, and the *x*-axis is the time in weeks. Followed left to right, each line tracks the proportion of movers as the interval grows from a common start. Although there are 9 U.S. Census divisions and 14 *régions* in Senegal, we calculate a total migration rate as the proportion of all individuals observed at a given start who changed location (division or *région)* over a given interval.

We are encouraged to report that the patterns that emerge from all three data sets are broadly similar and that our two expectations are generally confirmed. First, the level of migration observed increases as the interval increases. Although the trend is less obvious with a smaller buffer, it is still apparent. Second, increasing the buffer size both lowers the rates and reduces their multimodality with respect to time.

There is one obvious difference between these findings and our simulated results. In our model, the levels of migration estimated with a small buffer are consistently elevated, and the effect of increasing the buffer size is dramatic. Although we find some evidence of lower rates due to increases in the buffer size in the empirical data, smaller buffer estimates are not as consistently high. This discrepancy is partially due to the unrealistic assumption in the simulation of a uniform likelihood of short-term mobility. There are clear seasonal patterns, particularly in the Twitter estimates, that show when temporary mobility is more likely. If further simulation models are to be developed, more sophisticated strategies for modeling short-term mobility will be needed.

### Comparisons Across Data Sets

Given the differences in the contexts, the platforms, and the quality of the data in the three data sets, some interesting comparisons between them can be made. The smoothness of the estimates from Orange-Sonatel is surprising given that the administrative geography of the Senegalese *régions* is more fine-grained than that of the U.S. Census divisions. If short-term mobility is conditioned by distance, then we would expect to observe higher and more multimodal patterns of migration when the total distance an individual needs to cross a boundary is smaller. This is clearly not the case when we look at the results from Orange-Sonatel. Unlike the noisier estimates derived from Twitter and Gowalla, the Orange-Sonatel estimates lose their multimodal pattern as the buffer increases from one week to four weeks, likely because the regularity with which Orange-Sonatel users are observed makes it easier to remove the short-term mobility signal from the migration estimates.

The nearly four-year span of the Twitter data demonstrates that it is possible to analyze longer-term patterns and seasonal regularities. Across all three panels of Twitter estimates, it is apparent that there are seasonal bumps in short-term mobility. The consistency of these patterns is especially notable considering that the total number of Twitter users generating the data grew steadily over span of the data. A visual inspection of the broader trend in the Twitter data indicates that the relationship between migration level and the length of the interval is positive and almost linear, with the longest observed migration rates increasing as the interval grows, regardless of the choice of buffer size. This near-linear trend suggests little long-term *return* migration among our sample of Twitter users. As some individuals return to the place where they were first observed, we would expect the rate of increase with respect to interval to diminish. After all, survey and administrative data suggest that five-year migration estimates are almost always less than five times the corresponding one-year estimates (Rees 1977). In comparison, the smooth hump in the Orange-Sonatel estimates (Figure 4, top center and top right) resembles the pattern we would expect to observe as return migration diminishes the rate of increase, ultimately lowering the rate of migration observed with increased interval. Because the Orange-Sonatel data span just one calendar year, we can draw no conclusions about longer-term trends in the Senegalese context. However, results from this analysis show how short-term seasonal migration behavior in Senegal impacts the relationship between the migration estimate and the interval. Meanwhile, although the Gowalla estimates do not prove any additional insight per se, they do suggest that even sparse, low-quality digital trace data can contain an internally consistent migration signal.

**Fig. 5:**
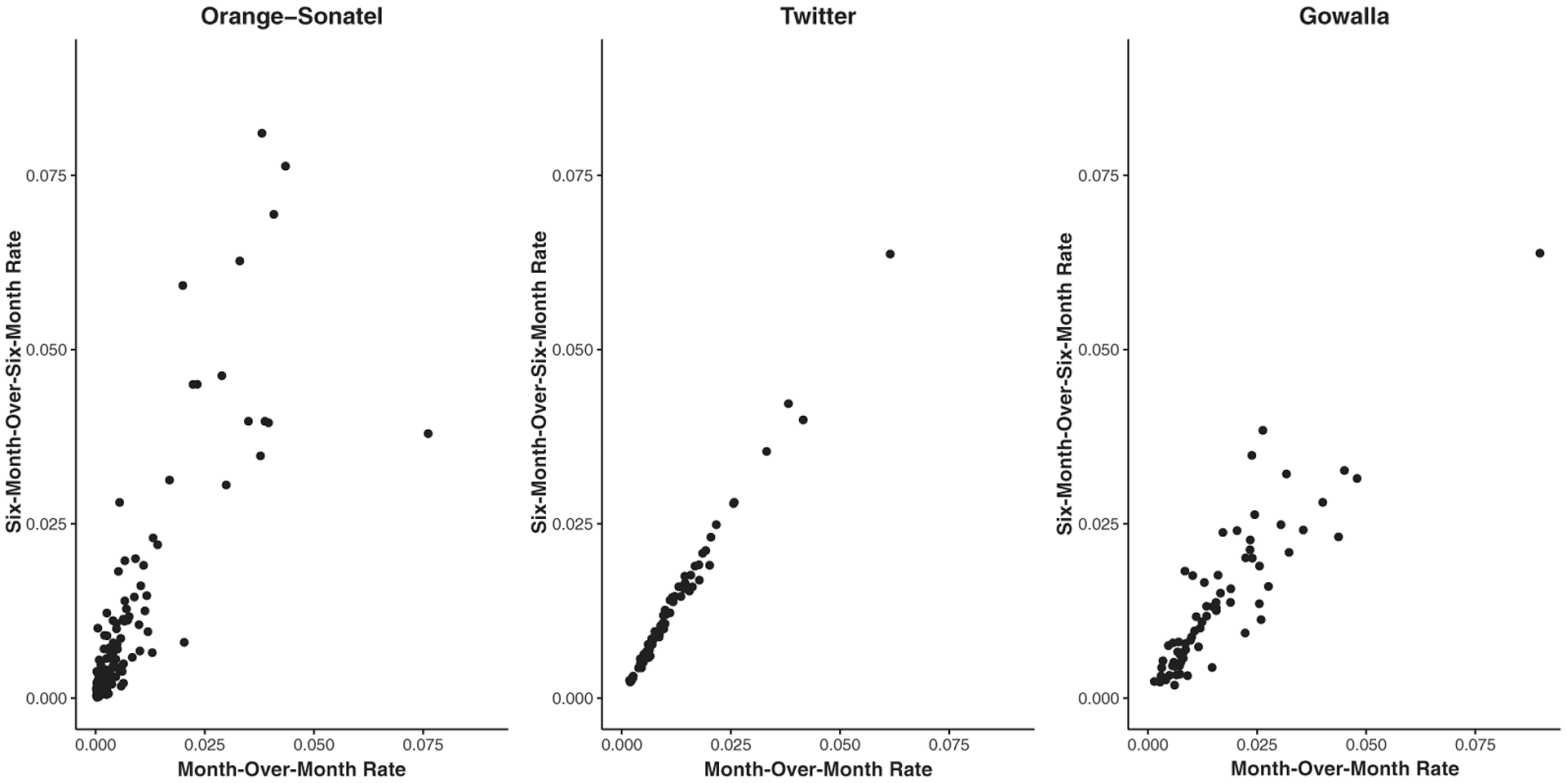
Each dot represents a bilateral flow or corridor, such as New England to the Mountain West (in the U.S. context). The migration rate corresponding to each bilateral flow is estimated at two different time scales: one-month-to-one-month and six-month-to-six-months. The plot shows the correlation of these two temporal specifications for each bilateral flow/corridor. The findings presented here provide fertile ground for future research and suggest that (easier to measure) short-term flows may be useful in modeling (harder to measure) long-term flows.

Figure 5 examines the geography of short-term and long-term mobility across the three data sets. Bilateral emigration rates are estimated for each possible pair of administrative units in the U.S. and Senegal contexts, respectively, using two temporal specifications: one month-over-month rate (e.g., place of residence in January vs. place of residence in February) and one six-month-over-six-month rate (e.g., place of residence from January to June vs. place of residence from July to December). This gives a short-term and a long-term estimate for 72 bilateral emigration flows in the United States (nine divisions × eight divisions) and for 182 bilateral emigration flows in Senegal (14 *régions* × 13 *régions).* When we plot the six-month rates against the one-month rates, we see a positive linear trend across all three data sets. If relatively few people in region *i* moved to region *j* over a one-month span, it is likely that relatively few people would have moved from *i* to *j* over a six-month span. Although this finding is preliminary, it suggests the novel possibility of modeling long-term rates between regions, which are harder to observe, using short-term rates. Our framework provides the conceptual flexibility necessary for investigating the relationship between short-term and long-term patterns of migration.

## Discussion and Next Steps

Migration data are expensive to collect, and different institutions use different temporal definitions of migration to meet their various needs. The harmonization of migration values estimated using different temporal definitions is difficult, which hampers the study of migration phenomena. These harmonization challenges arise in large part from complications due to return migration (i.e., the one-year/five-year problem) or temporary mobility (i.e., difficulties reconciling migration data defined by a different lengths of stay). Although our understanding of the temporal complexities of migration phenomena and measurement has historically been limited by scarce data, the availability of novel and increasingly prevalent forms of digital data creates opportunities for new research. In this paper, we have provided a series of methodological and theoretical advances that will make it easier to use digital trace data in migration research while leveraging their temporal granularity.

We developed a flexible method for converting georeferenced digital trace data, such as LBSN data or CDR data, into migration transition data. To address the growing number of *ad hoc* approaches in the literature, our method contributes a much-needed general terminology—*start, buffer, interval*—for this kind of inference procedure. We argue that these concepts refer to three distinct temporal dimensions of migration measurement: *start* measures seasonal- or period-specific effects; *buffer* measures residency criteria effects; and *interval* measures cumulative temporal effects. Because of the atomistic and indirect quality of digital trace data, all three concepts must be addressed to produce migration transition estimates, regardless of the quality or the geographic coverage of the data.

We then proposed an approach to assessing the quality and the characteristics of the migration signal present in a data source by systematically changing the temporal dimensions of the migration definition. In essence, this approach can be used to produce sets of estimates that track levels of migration along a quasi-continuous time scale analogous to a survival function. We applied this approach to simulated data as well as three empirical data sets from three platforms in two internal migration contexts. Our analysis addressed two research questions, premised on two hypotheses related to migration measurement.

First, we asked how measures of migration vary with respect to the time interval. Based on the literature on the one-year/five-year problem in migration measurement, we expected to find that migration estimates with larger intervals would be higher than the estimates with smaller intervals. Our simulation model illustrated this intuition, and our empirical analysis of three data sets confirmed it. At first blush, this finding may seem self-evident. However, because estimating migration along a pseudo-continuous time scale has never been done before, this result has important implications for further research. Now that we have demonstrated that migration rates increase as the interval increases, we can begin asking questions about the specific functional form of this relationship and about the extent to which it varies by geography or population characteristics. This approach also provides a means for assessing the temporal stability of migration estimates drawn from digital trace data. Technology platforms and their user bases are in constant flux. Any unexpected patterns with respect to the interval size should be treated as a red flag when they are found in specific data.

Second, we asked how measures of migration vary with respect to temporal residence criteria, which we called the *buffer.* Any research that uses digital trace data to measure migration will have to deal with questions about how to account for travel or temporary mobility, which may complicate the validity of migration estimates. We expected that migration data estimated with larger buffers would be less multimodal (i.e., noisy) than data estimated with smaller intervals. As in the case of our first research question, our simulation model illustrated this idea, and our empirical analysis confirmed it. Short-term travel is more common than long-term travel. Thus, as we increased the size of the buffer used to infer residency at either end of the interval, our migration estimates became smoother and more stable. Again, this finding may seem self-evident at first, but it demonstrates that by systematically varying the buffer, migration researchers can evaluate where the cut-off between travel and long-term relocation lies—a cutoff that will vary based on context and the purpose of the measurement. In our empirical analysis, we showed that the high degree of accuracy and the volume of CDR data from Orange-Sonatel makes it relatively easy to isolate long-term mobility from travel. As we increased the buffer from one week to four, the short-term spikes in migration disappeared from our plot. For the CDR data, we recommend using a buffer of at least four weeks to estimate migration. By contrast, the irregularity of the Twitter and Gowalla data made it more difficult to distinguish the migration signal from travel-related noise. Even when a buffer of 12 weeks was used, the plots from Twitter and Gowalla still exhibited some instability. For unprocessed social media data, we recommend using a buffer greater than 12 weeks to estimate migration. More research is needed to determine what the acceptable level of travel-related noise should be, but our method clearly provides a conceptual and methodological framework for systematically addressing this issue. Our findings point to an emergent research agenda using digital trace data to map the relationship between different kinds of migration estimates. Yet further work must be done to validate mobility and migration estimates by linking them to traditional data. Because our conceptual focus was on identifying *internal* consistencies within a series of migration estimates generated from the same data, we did not attempt to assess the *external* validity of these estimates. However, such an analysis is clearly needed. In the U.S. context, interdivisional migration rates from Twitter or Gowalla data could be linked to Internal Revenue Service or American Community Survey estimates. Overall, we are encouraged by other studies that have demonstrated the validity of migration estimates from digital trace data by linking them to traditional sources (e.g., Deville et al. 2014).

Additionally, more research is needed to confirm the fundamental results reported here. Although the simplicity of our framework and the intuitiveness of our findings suggest strong consistencies in how measurements of migration vary with the temporal definition, we tested for these consistencies using just three sets of data. Because the analysis presented here was limited to internal migration data, collecting and analyzing relevant data from international contexts in particular is essential. Moreover, research should be conducted to assess the robustness of the approach by leveraging migration corridors with well-known features. For example, flows between the United Kingdom and Spain certainly contain a very high proportion of travelers. Examining the sensitivity of international migration into and out of the United Kingdom estimated from digital trace data would both validate our method and characterize U.K. migration in- and out-flows in new and interesting ways.

## Footnotes

1 See https://www2.census.gov/geo/pdfs/maps-data/maps/reference/us_regdiv.pdf.

## References

[R1] Archive.org. (2016). Archive.org of Twitter 1% sample [Data set]. Retrieved from https://archive.org/details/twitterstream

[R2] Bell M (2004). Measuring temporary mobility: Dimensions and issues (Discussion Paper No. 2004/01). St. Lucia, Queensland, Australia: Queensland Centre for Population Research School of Geography.

[R3] Bell M, Charles-Edwards E, Kupiszewska D, Kupiszewski M, Stillwell J, & Zhu Y (2015). Internal migration data around the world: Assessing contemporary practice. Population, Space and Place, 21, 1–17.

[R4] Blumenstock JE, Gillick D, & Eagle N (2010). Who’s calling? Demographics of mobile phone use in Rwanda. Transportation, 32, 2–5.

[R5] Bojic I, Massaro E, Belyi A, Sobolevsky S, & Ratti C (2015). Choosing the right home location definition method for the given dataset. In Liu T-Y, Scollon CN, & Zhu W (Eds.), Social informatics (Proceedings of the 7th International Conference, Beijing, China, pp. 194–208). Cham, Switzerland: Springer.

[R6] Cassarino J (2004). Theorising return migration: The conceptual approach to return migrants revisited. International Journal on Multicultural Societies, 6, 253–279.

[R7] Cho E, Myers SA, & Leskovec J (2011). Friendship and mobility: User movement in location-based social networks. In Proceedings of the 17th ACM SIGKDD International Conference on Knowledge Discovery and Data Mining (pp. 1082–1090). New York, NY: Association for Computing Machinery.

[R8] DaVanzo J (1983). Repeat migration in the United States: Who moves back and who moves on? Review of Economics and Statistics, 65, 552–559.

[R9] de Beer J, Raymer J, van der Erf R, & van Wissen L (2010). Overcoming the problems of inconsistent international migration data: A new method applied to flows in Europe. European Journal of Population/Revue Européenne de Démographie, 26, 459–481. 21124647 10.1007/s10680-010-9220-z PMC2967706

[R10] de Montjoye Y, Smoreda Z, Trinquart R, Ziemlicki C, & Blondel VD (2014). D4D-Senegal: The second mobile phone data for development challenge (Unpublished manuscript). Retrieved from https://arxiv.org/abs/1407.4885

[R11] Deville P, Linard C, Martin S, Gilbert M, Stevens FR, Gaughan AE, … Tatem AJ (2014). Dynamic population mapping using mobile phone data. Proceedings of the National Academy of Sciences, 111, 15888–15893. 10.1073/pnas.1408439111 PMC4234567 25349388

[R12] Ellis M (2012). Reinventing US internal migration studies in the age of international migration. Population, Space and Place, 18, 196–208. 10.1002/psp.666 PMC4020137 24839406

[R13] Frias-Martinez V, Soto V, Hohwald H, & Frias-Martinez E (2012). Characterizing urban landscapes using geolocated tweets. In Proceedings of the ASE/IEEE 2012 International Conference on Social Computing and the 2012 International Conference on Privacy, Security, Risk and Trust (pp. 239–248). Washington, DC: IEEE.

[R14] Girardin F, Calabrese F, Dal Fiore F, Ratti C, & Blat J (2008). Digital footprinting: Uncovering tourists with user-generated content. IEEE Pervasive Computing, 7(4), 36–43.

[R15] Goldstein S (1964). The extent of repeated migration: An analysis based on the Danish population register. Journal of the American Statistical Association, 59, 1121–1132.

[R16] Gonzalez MC, Hidalgo CA, & Barabasi A-L (2008). Understanding individual human mobility patterns. Nature, 453, 779–782. 18528393 10.1038/nature06958

[R17] Gössling S, Ceron J-P, Dubois G, & Hall CM (2009). Hypermobile travellers. In Gossling S & Upham P (Eds.), Climate change and aviation: Issues, challenges and solutions (pp. 131–150). London, UK: Earthscan.

[R18] Greenberg I (1970). The first occurrence of n successes in N trials. Technometrics, 12, 627–634.

[R19] Haenszel W (1967). Concept, measurement, and data in migration analysis. Demography, 4, 253–261. 21279777 10.2307/2060366

[R20] Hannam K, Sheller M, & Urry J (2006). Mobilities, immobilities and moorings. Mobilities, 1, 1–22.

[R21] Hawelka B, Sitko I, Beinat E, Sobolevsky S, Kazakopoulos P, & Ratti C (2014). Geo-located twitter as proxy for global mobility patterns. Cartography and Geographic Information Science, 41, 260–271. 27019645 10.1080/15230406.2014.890072 PMC4786829

[R22] Jiang S, Ferreira J, & González MC (2017). Activity-based human mobility patterns inferred from mobile phone data: A case study of Singapore. IEEE Transactions on Big Data, 3, 208–219.

[R23] Jones M, & Pebley AR (2014). Redefining neighborhoods using common destinations: Social characteristics of activity spaces and home census tracts compared. Demography, 51, 727–752. 24719273 10.1007/s13524-014-0283-z PMC4048777

[R24] King R (1978). Return migration: A neglected aspect of population geography. Area, 10, 175–182.

[R25] King R, & Skeldon R (2010). Mind the gap! Integrating approaches to internal and international migration. Journal of Ethnic and Migration Studies, 36, 1619–1646.

[R26] Kitsul P, & Philipov D (1981). The one year/five year migration problem. In Rogers A (Ed.), Advances in multi regional demography (pp. 1–34). Laxenburg, Austria: International Institute for Applied Systems Analysis.

[R27] Long L, Tucker CJ, & Urton WL (1988). Migration distances: An international comparison. Demography, 25, 633–640. 3267544

[R28] Menchen-Trevino E (2013). Collecting vertical trace data: Big possibilities and big challenges for multi-method research. Policy & Internet, 5, 328–339.

[R29] Niedomysl T, Ernstson U, & Fransson U (2017). The accuracy of migration distance measures. Population, Space and Place, 23(1), e1971. 10.1002/psp.1971

[R30] Palmer JRB, Espenshade TJ, Bartumeus F, Chung CY, Ozgencil NE, & Li K (2013). New approaches to human mobility: Using mobile phones for demographic research. Demography, 50, 1105–1128. 23192393 10.1007/s13524-012-0175-z PMC3633623

[R31] Rees P (1977). The measurement of migration, from census data and other sources. Environment and Planning A: Economy and Space, 9, 247–272.

[R32] Rogers A (1995). Multiregional demography: Principles, methods and extensions. London, UK: Wiley.

[R33] Rogers A, Little J, & Raymer J (2010). The indirect estimation of migration: Methods for dealing with irregular, inadequate, and missing data. New York, NY: Springer.

[R34] Rogers A, Raymer J, & Newbold KB (2003). Reconciling and translating migration data collected over time intervals of differing widths. Annals of Regional Science, 37, 581–601.

[R35] Rogerson PA (1990). Migration analysis using data with time intervals of differing widths. Papers in Regional Science, 68, 97–106.

[R36] Roseman CC (1971). Migration as a spatial and temporal process. Annals of the Association of American Geographers, 61, 589–598.

[R37] Stock K (2018). Mining location from social media: A systematic review. Computers, Environment and Urban Systems, 71, 209–240.

[R38] Tasse D, Liu Z, Sciuto A, & Hong JI (2017). State of the geotags: Motivations and recent changes. In Proceedings of the Eleventh International AAAI Conference on Web and Social Media (ICWSM 2017) (pp. 250–259). Palo Alto, CA: AAAI Press.

[R39] Weber R, & Saarela J (2019). Circular migration in a context of free mobility: Evidence from linked population register data from Finland and Sweden. Population, Space and Place, 25(4), e2230. 10.1002/psp.2230

[R40] Williams AM, & Hall CM (2002). Tourism, migration, circulation and mobility: The contingencies of time and place. In Hall CM & Williams AM (Eds.), Tourism and migration: New relationships between production and consumption (pp. 1–52). Dordrecht, the Netherlands: Springer.

